# An Immune Assay to Quantify the Neutralization of Oxidation-Specific Epitopes by Human Blood Plasma

**DOI:** 10.3390/antiox14080903

**Published:** 2025-07-24

**Authors:** Marija Jelic, Philipp Jokesch, Olga Oskolkova, Gernot Faustmann, Brigitte M. Winklhofer-Roob, Bernd Ullrich, Jürgen Krauss, Rudolf Übelhart, Bernd Gesslbauer, Valery Bochkov

**Affiliations:** 1 Department of Pharmaceutical Chemistry, Institute of Pharmaceutical Sciences, University of Graz, 8010 Graz, Austriao.oskolkova@uni-graz.at (O.O.);; 2 Institute of Molecular Biosciences, University of Graz, 8010 Graz, Austria; 3 Heidelberg ImmunoTherapeutics GmbH, 69115 Heidelberg, Germany; 4 Vanudis GmbH, 69115 Heidelberg, Germany; 5 Field of Excellence BioHealth, University of Graz, 8010 Graz, Austria

**Keywords:** oxidized phospholipids, OxLDL, oxidation-specific epitopes, ELISA

## Abstract

Oxidized phospholipids (OxPLs) are increasingly recognized as biologically active lipids involved in various pathologies. Both exposure to pathogenic factors and the efficacy of protective mechanisms are critical to disease development. In this study, we characterized an immunoassay that quantified the total capacity of the plasma to degrade or mask OxPLs, thereby preventing their interaction with cells and soluble proteins. OxLDL-coated plates were first incubated with human blood plasma or a control vehicle, followed by an ELISA using a monoclonal antibody specific to oxidized phosphatidylethanolamine. Pretreatment with the diluted blood plasma markedly inhibited mAb binding. The masking assay was optimized by evaluating the buffer composition, the compatibility with various anticoagulants, potential interfering compounds, the kinetic parameters, pre-analytical stability, statistical robustness, and intra- and inter-individual variability. We propose that this masking assay provides a simple immunological approach to assessing protective mechanisms against lipid peroxidation products. Establishing this robust and reproducible method is essential for conducting clinical association studies that explore masking activity as a potential biomarker of the predisposition to a broad range of lipid-peroxidation-related diseases.

## 1. Introduction

Oxidation-specific epitopes (OSEs) are produced through the modification of biopolymers by lipid peroxidation products (LPPs) [1]. Changes in the functional activity of proteins induced by covalent modifications are thought to be a major pathogenic mechanism of oxidative stress. One type of LPP that gives rise to OSEs is oxidized phospholipids (OxPLs), which are increasingly recognized as biologically active lipids present both in free forms and as protein adducts [2,3]. The best-characterized classes of OxPLs are oxidized phosphatidylcholines (OxPCs) and phosphatidylethanolamines (OxPEs), which accumulate in various tissues and disease states and exhibit a range of biological activities. Potentially important for atherogenesis is the involvement of OxPLs in foam cell formation, chemokine production, and platelet activation and coagulation [2,4,5,6]. The importance of endogenously generated OxPCs in disease has been documented in several types of cardiovascular and liver pathology, as well as in ischemia–reperfusion injury and osteoporosis [7,8,9,10]. In summary, OxPLs are increasingly recognized as damage-associated molecular patterns (DAMPs) actively involved in disease pathogenesis [11].

Accumulating evidence on the pathogenic importance of OxPLs underscores the need to investigate the mechanisms of their neutralization. Apart from cleavage by lipoprotein-associated phospholipase A_2_ [12], the best-characterized mechanism is mediation by immunoglobulins of the M class (IgM). OSE-specific IgM is highly abundant in the plasma and makes up a significant portion of the total circulating IgM [13]. Three potential mechanisms of antigen inactivation by IgM are known. IgM can either stimulate OSE clearance through Fc-receptor (FcR) and complement-receptor-mediated endocytosis or neutralize OSEs by blocking interactions with proteins that induce pathological effects such as scavenger receptors [14]. In the context of atherosclerosis, inactivation of the first two mechanisms (FcR or complement C1q subcomponent (C1q)) has minimal effects on atherogenesis [15,16]. In contrast, the importance of the antigen neutralization mechanism has been supported by data showing that the single-chain variable fragment of an OxPC-specific antibody E06-scFv, which does not bind to FcR or C1q but is capable of binding to OxPLs and inhibiting OxLDL uptake by scavenger receptors, demonstrated protective effects in atherogenesis and other conditions [7]. These data suggest that antibody binding hinders the recognition of OxPC by scavenger and other pro-atherogenic receptors and thereby prevents the pathogenic effects of this OSE [14].

IgM is an important class but not the only class of soluble OxPL-binding proteins that is present in the plasma. Based on genetic and biochemical data, C-reactive protein (CRP) and complement factor H (CFH) have been suggested as potential OxPC-binding proteins, a finding that was confirmed by binding assays [17,18]. Recently, we conducted a search for OxPC-binding plasma proteins using pulldown proteomics with liposomes containing oxidized palmitoyl-arachidonoyl-phosphatidylcholine (OxPAPC) and identified additional plasma proteins capable of binding OxPLs [19]. We hypothesize that these proteins can at least partially act analogously to natural IgM antibodies, i.e., neutralize OxPCs by sterically preventing their recognition by pro-inflammatory and pro-atherogenic receptors. It is tempting to speculate that in addition to the bulk concentration of OSEs accumulating in the blood and tissues, the activity of OSE-neutralizing mechanisms also influences the risk of developing atherosclerosis and other pathologies. Importantly, the neutralizing activity of an individual’s plasma is expected to result from the combined action of multiple OSE-binding and OSE-degrading proteins. Because human plasma contains a variety of OSE-recognizing proteins, quantifying each individually—such as through multiple ELISA tests—is impractical. Instead, a simple assay to measure the total OSE-binding/neutralizing activity of individual plasma samples is needed to facilitate the analysis of the clinical associations between the total OSE-neutralizing activity in the plasma and disease. We previously described a pilot version of such an assay [20], which was based on the ability of plasma proteins to mask OSEs on the surface of OxLDL, preventing recognition by OxPL-specific monoclonal antibodies. Our pilot clinical study demonstrated reduced masking activity in patients with hypertension, coronary artery disease, acute coronary syndrome, and type 2 diabetes mellitus [20]. To emphasize that, in addition to antibodies, non-immunoglobulin proteins also bind to or cleave OxPLs, we coined the term “OSE-masking assay” to distinguish it from “antigen neutralization”, which specifically describes the action of antibodies.

The objective of this study was to optimize the masking procedure to establish a reliable and reproducible assay for screening large cohorts of patients and control subjects. To this end, the study investigates key biochemical and statistical parameters of the masking assay, including the buffer composition, compatibility with various anticoagulants, potential interfering compounds, kinetic properties, pre-analytical stability, statistical robustness, and intra- and inter-individual variability. The ultimate goal is to develop a method for estimating individual risk of lipid-peroxidation-related diseases.

## 2. Materials and Methods

### 2.1. The Materials and Reagents

The Nunc MaxiSorp plates (Cat. No 439454) were from Thermo Fisher Scientific (Waltham, MA, USA). The mouse monoclonal antibody (mAb 509, [20]) was prepared by concentrating hybridoma culture fluid (UltraDOMA Protein-free Medium, Lonza, Basel, Switzerland) ten-fold using centrifugal ultrafiltration cartridges (Amicon Ultra-4, a 100 kDa cutoff, Merck Millipore (Burlington, MA, USA), Cat. No UFC810024). The concentration of mAb 509 was determined using a mouse IgM ELISA kit (Thermo Fisher Scientific, Cat. No 22-156-777). The monoclonal antibody clone 1605 against human apoB was from Santa-Cruz (Santa Cruz, CA, USA), Cat. No sc-58237. The control mouse IgM produced using the MOPC104E myeloma cell line was from Merck (Darmstadt, Germany), Cat. No M5909. The protease inhibitor cocktails were from Merck (Cat. No 11836145001) and Thermo Fisher Scientific (Cat. No A32955). OxLDL, ortho-phenylenediamine, and the stable peroxide substrate buffer were from Thermo Fisher Scientific (Cat. Nos L34357, 34005, and 34062). The anti-mouse IgM HRP conjugate was from Abcam, Cambridge, UK (Cat. No AB97230). The other reagents were purchased from Merck.

### 2.2. Human Plasma

Fresh plasma samples were collected from volunteers using K_3_EDTA blood collection tubes (Vacuette, Greiner Bio-One, Kremsmünster, Austria). The samples were pooled, aliquoted, snap-frozen in liquid nitrogen, and stored at −80 °C until use.

#### 2.2.1. Blood Sampling from Healthy Volunteers and COVID-19 Patients

Blood samples were obtained from SARS-CoV-2-seronegative subjects, hospitalized patients with acute COVID-19 disease, and recovered patients with asymptomatic, mild, or severe COVID-19 infections within a clinical non-AMG study, which was conducted at four clinical sites in Heidelberg, Koblenz, and Berlin and approved by the Ethics Committees of Heidelberg (S-719/2020), LÄK Rheinland-Pfalz (2021-16111), and Berlin (Eth-29/21) (all in Germany). This clinical study was supported by Heidelberg ImmunoTherapeutics GmbH.

#### 2.2.2. Blood Sampling for the Day-to-Day Variability Study

The day-to-day variability analysis was performed using samples from the BIOmarkers of Robustness of Metabolic Homeostasis for Nutrigenomics-derived Health CLAIMS Made on Food (BIOCLAIMS) project, funded by the Seventh Framework Programme of the European Commission [21], which included a day-to-day variability study performed at the University of Graz, Austria, and the Medical University of Graz, Austria, in 2015 [22], along with sample collection for collaborative biomarker research. Approval for the BIOCLAIMS day-to-day variability study was obtained from the Ethics Committees of the Medical University of Graz (EK 23-306 ex 10/11; approval date: November 24, 2014) and the University of Graz (GZ.39/45/63 ex 2014/15; confirmation date: March 31, 2015), and a Sample Transfer Agreement was put in place in 2025.

Blood samples from 7 healthy volunteers were collected over five consecutive days (Monday to Friday of a given week), following an overnight fast. Samples were drawn in the mornings at similar time points on each of the five days. The study subjects were advised to avoid changes in their physical activity and dietary intake across the 5 days. Blood was collected into EDTA-coated tubes (Vacuette, Greiner Bio-One) and centrifuged immediately after venipuncture, and plasma aliquots were frozen within 30 min. The samples were stored at −80 °C under controlled conditions from 2015 to 2024 until the analysis.

### 2.3. The Standard Masking Assay

In brief, 96-well microtiter plates were coated with OxLDL (7.5 µg/mL) in 50 µL/well of phosphate-buffered saline containing 0.5 mM EDTA (PBSE) overnight at 4 °C. Phosphate-buffered saline (PBS) was prepared as a 10 × stock containing the following salts: 1.37 M NaCl (80 g/L), 0.0268 M KCl (2 g/L), 0.176 M KH_2_PO_4_ (24 g/L), and 0.081 M Na_2_HPO_4_ · 2H_2_O (14.4 g/L). After 10-fold dilution of the 10 × PBS, EDTA was added from a 0.5 M Na-EDTA stock, with a pH of 8.0, to the final concentration of 0.5 mM. The pH of the 1 × PBSE buffer was 7.4; no pH adjustment was required after the dilution and addition of EDTA. On the next day, the plates were washed 4 times with 200 µL/well of PBSE and blocked for 1 h at 23 °C with 100 µL/well of fish gelatin (30 mg/mL). After washing with PBSE, 50 µL/well of human plasma diluted 1:20 in PBSE with bovine serum albumin (BSA) (10 mg/mL) was incubated for 1 h at 37 °C. The plate was washed again and filled with 50 µL/well of mAb 509 (10 µg/mL) diluted in PBSE/BSA (10 mg/mL). After incubation at 23 °C for 1 h, the plate was washed 4 times with 200 µL of PBSE and incubated with 50 µL/well of the anti-mouse IgM HRP conjugate (1:3000) at 23 °C for 1 h. The plate was washed 4 times with 200 µL of PBSE before the addition of 50 µL/well of the ortho-phenylenediamine (0.5 mM)/peroxide substrate mixture. After 15 min at 23°, the reaction was stopped by the addition of 50 µL/well of 2 M H_2_SO_4_, followed by measurement of the optical density at 492 nm. All incubations were performed with 4 to 6 replicates.

### 2.4. The Statistical Analysis

All of the data are presented as means ± SD. The statistical analysis was conducted in the GraphPad Prism program (version 10.4.2) using a one-way ANOVA with post hoc Tukey’s multiple comparisons test. *p* values of less than 0.05 were considered statistically significant: * *p* ≤ 0.05, ** *p* ≤ 0.01, and *** *p* ≤ 0.001.

## 3. Results

### 3.1. Optimization of the OSE-Masking ELISA Procedure

#### 3.1.1. The Composition and pH of the Buffer

The key step of the assay was incubation of the microtiter-plate-immobilized OxLDL with diluted plasma (1:20–1:40). We performed dilutions using PBS, which is a common laboratory buffer maintaining a pH and ionic strength close to physiological values. A practical advantage of PBS is that it can be prepared as a 10-fold stock solution and, after dilution, be used without pH adjustments (see the “Methods” section). To prevent further peroxidation of OxLDL via the Fenton reaction catalyzed by Fe^2^⁺ and Cu^2^⁺ ions, PBS was supplemented with 0.5 mM of EDTA throughout the procedure.

**Figure 1:**
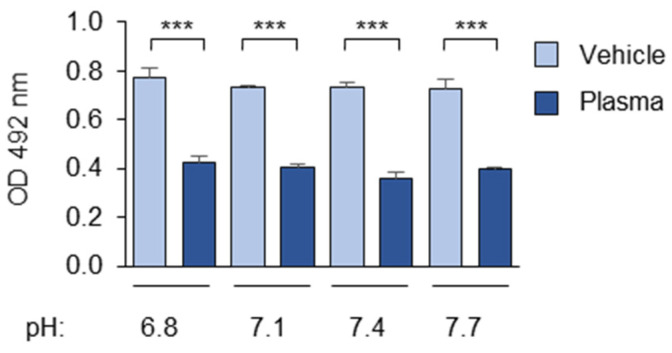
The effect of near-physiological pH variations on the masking activity. Incubation with 1:20-diluted pooled blood plasma was performed in PBSE/BSA (10 mg/mL) with the pH adjusted to the indicated values by changing the ratio of Na_2_HPO_4_/NaH_2_PO_4_ in the PBSE. Further incubations with mAb 509, the anti-mouse IgM conjugate, and the substrates were performed at a pH of 7.4 according to the standard conditions described in the “Methods” section. *** *p* ≤ 0.001.

We found that variations in the pH value within the near-physiological range from 6.8 to 7.7 had no significant influence on masking (Figure 1).

#### 3.1.2. Blocking Proteins

The plates were blocked after coating them with OxLDL using fish gelatin (30 mg/mL) instead of commonly used BSA in order to prevent contamination with the mammalian OSE potentially present in blood-derived BSA. After blocking, the wells were treated with plasma diluted in PBSE/BSA (10 mg/mL) or a vehicle control (PBSE/BSA (10 mg/mL) without plasma). The masking activity was calculated through normalization of the optical density in the plasma-treated wells to the control values.

**Figure 2:**
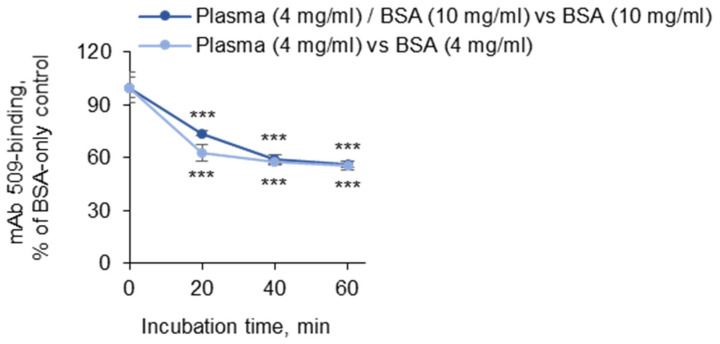
A comparison of two BSA concentrations for determination of the masking effect. Coating of the plates with OxLDL and blocking with 3% fish gelatin were performed according to the standard procedures. After washing, the wells were filled with 50 µL of PBSE/BSA (10 mg/mL). Then, 50 µL aliquots of plasma diluted 1:10 (2-fold-concentrated as compared to that in the standard procedure) were added to the 50 µL already present in the plate, thus resulting in final dilution of 1:20. Plasma was added at time 0 and after 20, 40, and 60 min, thus resulting in incubation times of 60, 40, 20, and 0 min. The experiment was performed under four conditions within the same plate: (a) BSA (10 mg/mL); (b) BSA (10 mg/mL) + plasma (1:20); (c) BSA (4 mg/mL); and (d) plasma (1:20), with no BSA. Because the pooled plasma contained 80 mg/mL of protein, the total protein concentrations in samples (c) and (d) were identical. After the last addition of plasma at 60 min, the plate was immediately washed and treated with mAb 509, the anti-mouse IgM conjugate, and substrates according to the standard conditions described in the “Methods” section. The two curves represent the ratios of samples (b) to (a) and (d) to (c) at the indicated time points. Note that different BSA concentrations did not significantly change the kinetics or maximal values of the masking activity. Statistical significance is shown as compared to time point 0. *** *p* ≤ 0.001.

It was found that the residual (i.e., control-normalized) binding of mAb 509 after pretreatment with the plasma reached saturation after 20–40 min of incubation (Figure 2, dark symbols). Similar kinetics was observed when OxLDL was incubated with plasma (4 mg/mL plasma protein) in PBSE without BSA, and the binding was normalized to that after treatment with an equal concentration of the blocking protein without plasma (PBSE/BSA (4 mg/mL)); see Figure 2, light blue symbols. The data show that the total concentration of BSA does not significantly change the kinetics or the maximal degree of masking induced by the plasma.

**Figure 3:**
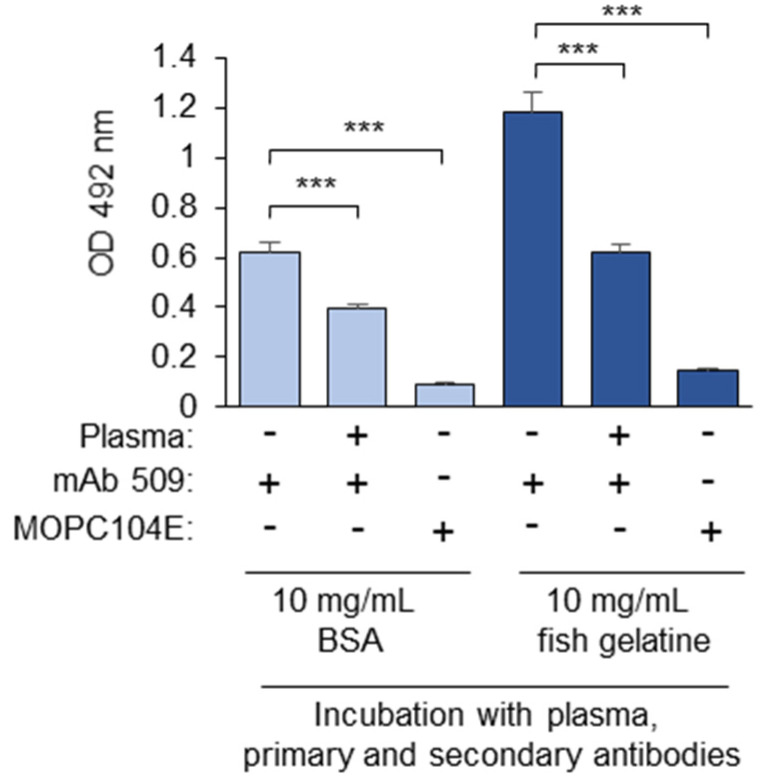
A comparison of BSA and fish gelatin as the blocking proteins in the masking assay. Coating the plates with OxLDL and blocking with fish gelatin (30 mg/mL) were performed according to the standard procedures. Further incubations with plasma, mAb 509, MOPC104E, and the anti-mouse IgM conjugate were performed either in the presence of BSA (10 mg/mL) or fish gelatin (10 mg/mL). *** *p* ≤ 0.001.

Fish gelatin (10 mg/mL) could also be used throughout the masking assay, including blocking and incubation with plasma and the first and secondary antibodies. In this case, the absolute OD values were higher, but the non-specific binding of an isotype control IgM (MOPC104E) was also increased (Figure 3). As a result, the calculated ratios are comparable: 0.53 in the BSA-blocked samples and 0.46 in the gelatin-blocked samples. Moreover, the assay is intended for use in cohort studies, in which both patient and control samples are processed using the same blocking protein. If not stated otherwise, in further experiments, the incubation with plasma and the antibodies was performed in the presence of PBSE/BSA (10 mg/mL). This concentration significantly exceeds the protein concentration in diluted blood plasma, thereby minimizing the potential impact of inter-individual variations in human serum albumin levels, which may be important for clinical cohort studies.

#### 3.1.3. Washing

The washing steps were performed using PBSE without protein. Detergents commonly applied for washing (e.g., Tween 20) were avoided because of potential solubilization of lipophilic OSEs, such as free OxPLs.

#### 3.1.4. Incubation Times

**Figure 4:**
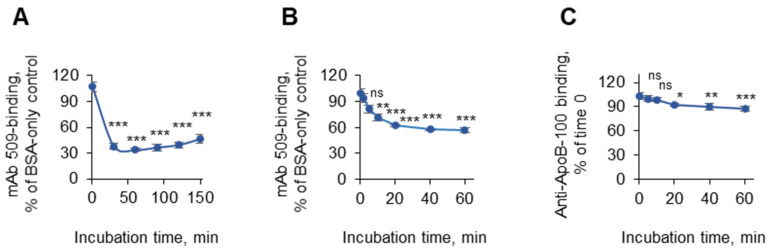
The time kinetics of the masking effect. Coating of the plates with OxLDL and blocking with fish gelatin (30 mg/mL) were performed according to the standard procedures. After washing, the wells were filled with 50 µL of PBSE/BSA (10 mg/mL). (**A**) Plasma was diluted 1:10 (2-fold-concentrated as compared to the standard procedure) in the same solution and stored on ice. Then, 50 µL aliquots of diluted plasma were added at different times to the 50 µL already present in each well to obtain incubation times of 150, 120, 90, 60, 30, and 0 min at 1:20 plasma dilution. In panel (**B**), all incubations were performed in the presence of fish gelatin instead of BSA, and plasma incubation times of 0, 5, 10, 20, 40, and 60 min were used. At the end of incubation, all wells were simultaneously washed and treated with mAb 509, the anti-mouse IgM conjugate, and the substrates according to the standard conditions described in the “Methods” section. In panel (**C**), the OxLDL-coated wells were incubated for different times with plasma (1:20) in PBSE/BSA (10 mg/mL). The ELISA in (**C**) was performed using an anti-apoB mAb (Santa Cruz). Statistical significance is shown as compared to time point 0. ns—non-significant, * *p* ≤ 0.05, ** *p* ≤ 0.01, and *** *p* ≤ 0.001.

We further determined the time kinetics of masking. It was found that at 37 °C, the masking reached a plateau value within 20 min after the addition of plasma diluted in PBSE/BSA (10 mg/mL); this constant level was stable for more than 2 h (Figure 4A). Similar kinetics was observed when PBSE/fish gelatin (10 mg/mL) was used for incubation with antibodies (Figure 4B). To ensure that the masking effect was not caused by the washing-off of entire OxLDL particles from the plastic, we repeated the experiment using anti-apoB instead of mAb 509 and observed a minimal reduction in the apoB immunoreactivity after 60 min of incubation, thus showing that the majority of the lipoprotein remained attached to the plastic (Figure 4C).

#### 3.1.5. Optimization of the Antigen and Antibody Concentrations

**Figure 5:**
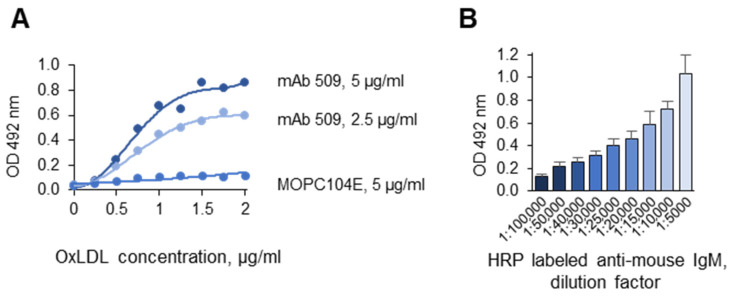
Titration with (**A**) OxLDL, mAb 509, and (**B**) the anti-mouse IgM HRP conjugate. Experiments were performed according to the standard procedures described in the “Methods”, except that different concentrations of OxLDL and mAb 509 (**A**) or the anti-mouse IgM HRP conjugate (**B**) were used.

The signal intensity of the assay depended on the concentrations of OxLDL and of the first and secondary antibodies. The results of titration with OxLDL, mAb 509, and anti-mouse IgM-HRP are presented in Figure 5A and Figure 5B, respectively. In further experiments, higher concentrations of mAb 509 (10 µg/mL) and secondary antibodies (1:3000) were used to ensure excess detection reagents over the amount of the antigen.

#### 3.1.6. Anticoagulants

**Figure 6:**
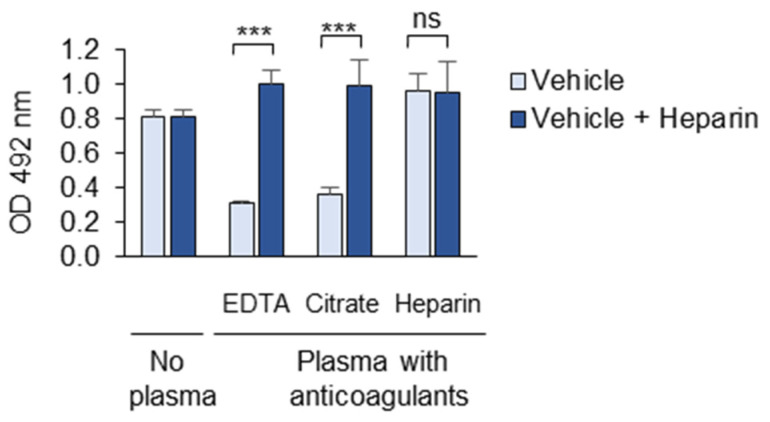
The effects of anticoagulants on the masking activity. The experiment compared the masking activities of plasma samples collected from the same donor in EDTA, citrate, and heparin anticoagulants. Such triple samples were obtained from three donors and pooled. The experiment was performed according to the standard procedure described in the “Methods” section. Where indicated, heparin (20 µg/mL) was added during the incubation of the plasma with immobilized OxLDL. ns—non-significant, *** *p* ≤ 0.001.

We further compared the masking activity of plasma collected from the same donor in different anticoagulants, namely EDTA, citrate, and heparin. Such triple samples were obtained from three donors and combined to make EDTA-, citrate- and heparin-anticoagulated pools. The plasma samples anticoagulated with the divalent ion chelators EDTA and citrate were active and demonstrated comparable masking effects (Figure 6). In contrast, the heparin-anticoagulated plasma showed no masking activity (Figure 6). The addition of heparin (20 µg/mL) to the EDTA- and citrate-anticoagulated plasma completely reversed the masking effect.

#### 3.1.7. Polyanions and Polycations Interfere with the Masking Assay

**Figure 7:**
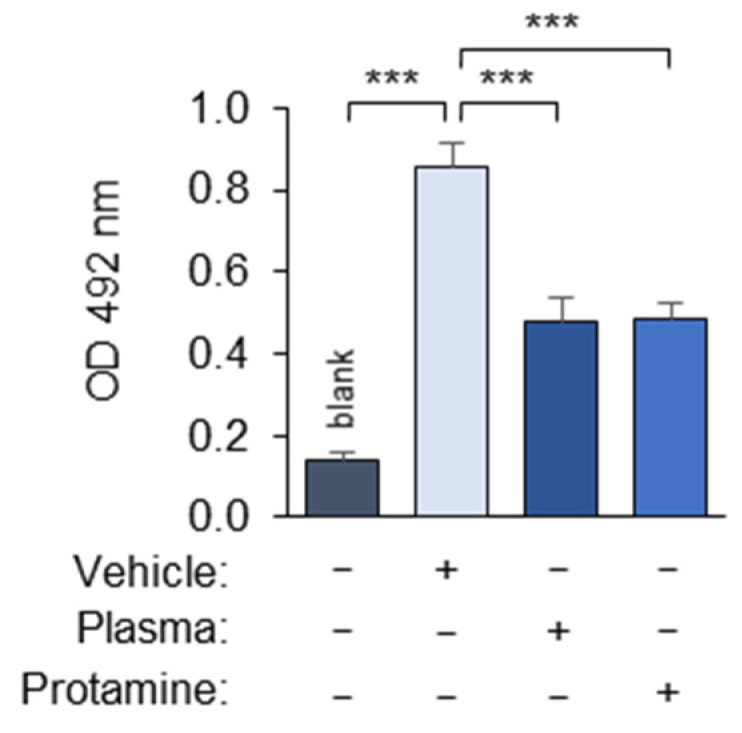
Protamine demonstrates masking activity. The experiment was performed according to the standard procedure described in the “Methods” section in the absence or presence of polycation protamine, which was added during the incubation of OxLDL with the plasma. Blank values were obtained by omitting mAb 509. PBSE/BSA (10 mg/mL) was used as a vehicle. *** *p* ≤ 0.001.

The addition of the polyanion heparin to the EDTA- and citrate-anticoagulated plasma completely reversed the masking effect (Figure 6). In contrast, a small (5 kDa) positively charged protein, protamine, induced masking in the absence of the plasma (Figure 7). The masking activity of protamine serves as important proof of principle that the interaction of the proteins with OxLDL can reduce its recognition by mAb 509. In summary, the data show that the masking activity of the plasma is strongly influenced by polyanions and polycations, and therefore, highly charged polymers should be avoided in the assay. All of the experiments described in this manuscript were performed using EDTA-anticoagulated human blood plasma.

**Figure 8:**
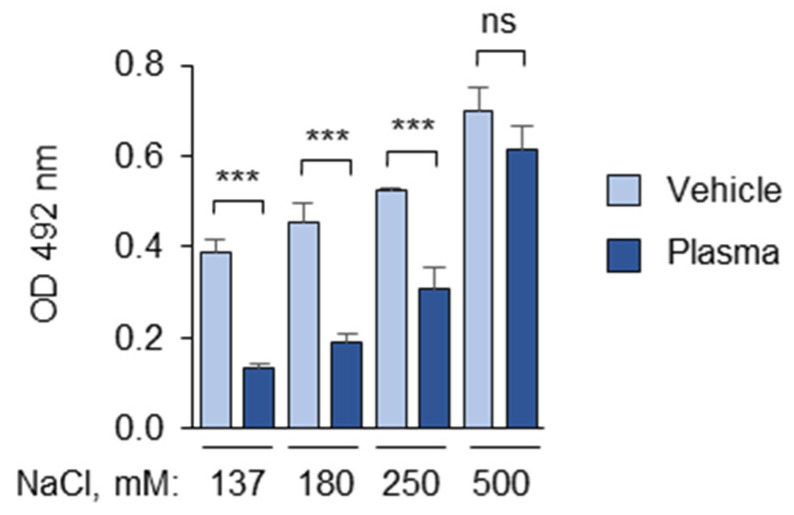
A high concentration of NaCl inhibits the masking activity. The experiment was performed according to the standard procedure described in the “Methods” section in the presence of the indicated final concentrations of NaCl (including 137 mM NaCl in PBSE) during the incubation of OxLDL with the plasma. Dry salt was added to PBSE to obtain the NaCl concentrations indicated. ns—non-significant, *** *p* ≤ 0.001.

The interfering effects of the polyanions and polycations underscore the importance of ionic interactions in the development of the masking effect. To inhibit these interactions, the ionic strength of the incubation medium was increased by adding NaCl. It was observed that increasing the NaCl concentrations in the diluted plasma during incubation with OxLDL significantly inhibited the masking activity but only at non-physiological NaCl levels (250 mM or higher, including 137 mM NaCl in PBS) (Figure 8).

#### 3.1.8. Laboratory Reagents Interfere with the Masking Assay

**Figure 9:**
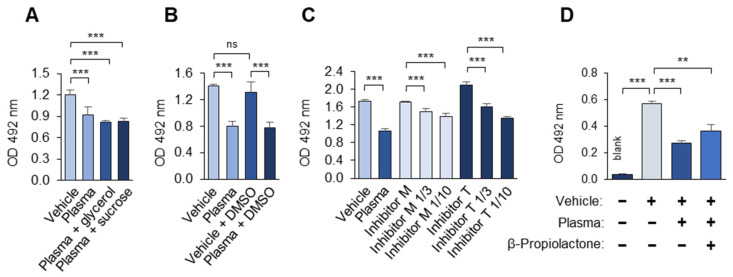
The effects of common laboratory reagents on the masking activity. The experiment was performed according to the standard procedure described in the “Methods” section, except that the incubation of the plasma with OxLDL was performed in the presence of (**A**) glycerol (10% *w*/*v*) or sucrose (10% *w*/*v*), (**B**) DMSO (0.5% *v*/*v*), (**C**) protease inhibitor cocktails from Merck and Thermo Fisher Scientific at the concentrations recommended by the manufacturers and diluted 3- and 10-fold, and (**D**) β-propiolactone (0.1%). Blank values were obtained by omitting mAb 509. ns—non-significant, ** *p* ≤ 0.01 *** *p* ≤ 0.001.

Finally, the effects of several common laboratory reagents on the masking activity were tested. It was found that the low concentrations of DMSO that are used for the solubilization of pharmacological substances, as well as glycerol and sucrose (often applied for protein stabilization), did not interfere with the masking activity (Figure 9A,B). In contrast, protease inhibitor cocktails from two manufacturers strongly inhibited masking (Figure 9C). Furthermore, β-propiolactone, which is widely used for the inactivation of viruses and other pathogens, also suppressed the masking activity (Figure 9D). Thus, this method cannot be used to analyze plasma samples that contain protease inhibitor cocktails or that have been treated with β-propiolactone, and probably other reactive electrophiles.

#### 3.1.9. The Stability of the OSE-Masking Activity During the Pre-Analytical Procedures

**Figure 10:**
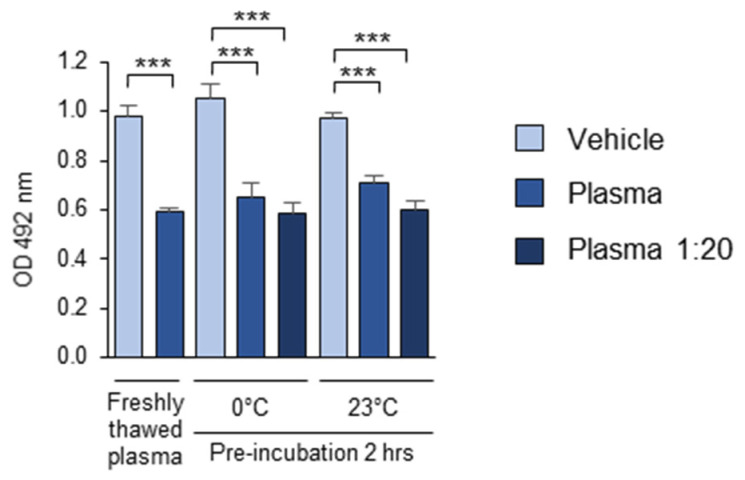
The pre-analytical stability of the masking activity. The experiment was performed according to the standard procedure described in the “Methods” section, except that the plasma samples were thawed and stored for 2 h on ice (0 °C) or at room t° (23 °C) either undiluted or diluted 1:20 in PBSE/BSA (10 mg/mL). Control PBSE/BSA (10 mg/mL) solutions were incubated under the same conditions. *** *p* ≤ 0.001.

The multiple plasma samples used in this study were stored at −80 °C, suggesting that this standard plasma storage method can be effectively applied in OSE-masking studies. The short-term stability of the OSE-masking activity in the plasma during the pre-analytical procedures was assessed by comparing the activity of freshly thawed pooled plasma with another aliquot of the same plasma, thawed 2 h in advance and stored on ice or at room temperature before being added to the OxLDL-coated wells. Furthermore, to determine whether plasma dilution affected the stability of the masking activity, additional plasma samples were diluted 1:20 with PBSE and incubated for 2 h on ice or at room temperature. Plasma samples thawed and diluted immediately before their addition to OxLDL served as the controls. The results showed that the masking activity of both the undiluted and 1:20-diluted plasma remained stable for at least 2 h, whether stored on ice (0 °C) or at room temperature (23 °C) (Figure 10). These findings suggest that the standard short-term storage of thawed plasma samples under cooling does not significantly alter their OSE-masking activity.

#### 3.1.10. Optimization of the Plasma Dilution

**Figure 11:**
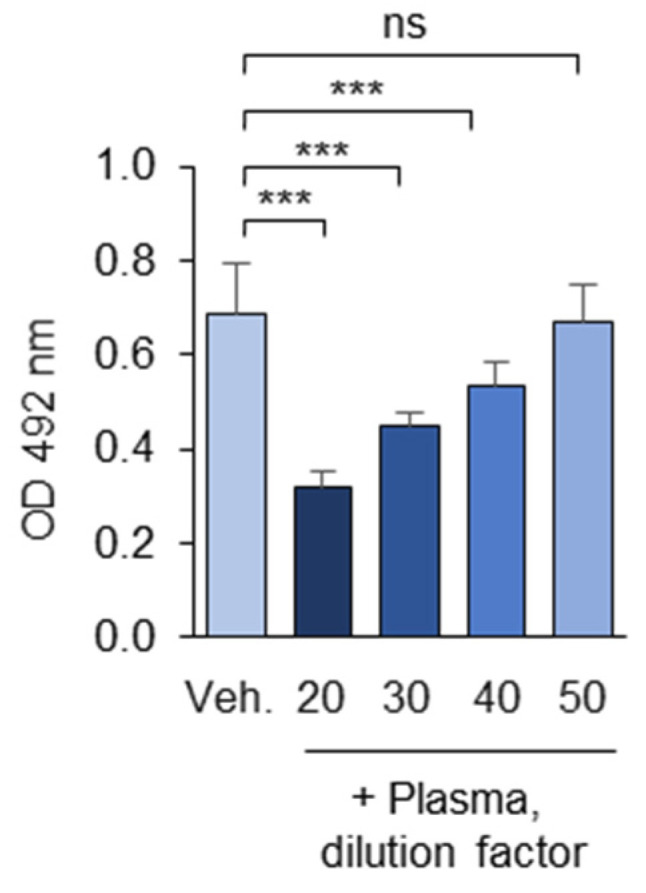
Concentration dependence of masking activity. The experiment was performed according to the standard procedure described in the “Methods” section, except that different plasma dilutions were used. ns—non-significant, *** *p* ≤ 0.001.

The OSE-masking effect was dependent on the plasma dilution (Figure 11). Dilutions below 1:20 (5% *v*/*v*) were avoided due to the high concentration of plasma albumin, which could potentially induce non-specific signal reductions. For cohort studies, intermediate plasma dilution rates (e.g., 1:35) can be used to identify subjects with either high or low OSE-masking activity.

#### 3.1.11. Determination of Non-Specific Binding

**Figure 12:**
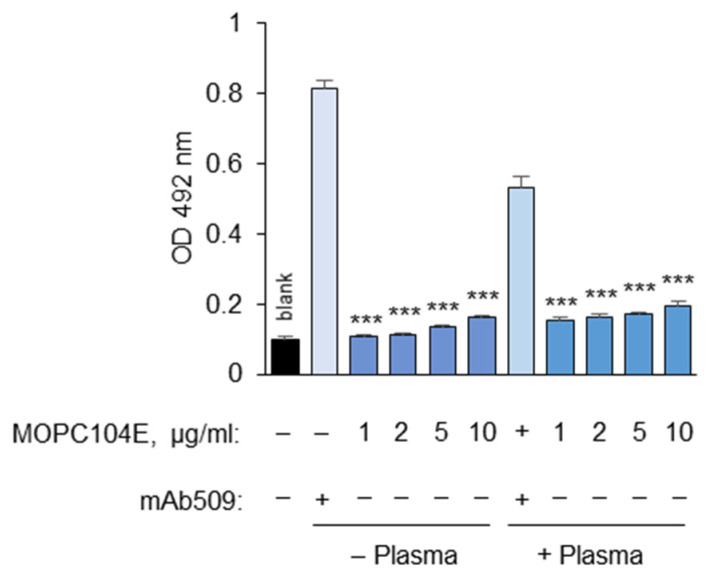
The determination of non-specific binding using the mouse IgM control mAb MOPC104E. mAb 509 was used at 10 µg/mL. The left column presents the no-first-antibody blank value. It can be seen that IgM has some affinity with OxLDL, and the resulting OD492 values tend to be higher than those for the no-first-antibody control, especially after pretreatment of the OxLDL with plasma. The data suggest that MOPC104E may be a better control in the masking assay as compared to the no-first-antibody control. Statistical significance is shown as compared to the mAb 509 values either with or without plasma pretreatment. *** *p* ≤ 0.001.

As a control for non-specific binding of primary mAbs to plastic-immobilized OxLDL, we used the commercially available mouse IgM isotype control MOPC104E, for which no antigen is known. We observed the trend of higher non-specific binding with the MOPC104E isotype control compared to that for the no-mAb 509 control (Figure 12). This non-specific binding increased further after the plasma treatment (Figure 12). Therefore, it is recommended to assess non-specific binding using the mouse IgM control rather than relying on no-antigen or no-primary-antibody blank values. Importantly, other commercially available isotype control IgMs may interact with OxLDL much more strongly than MOPC104E. For example, we observed significant binding of MOPC21 to OxLDL.

### 3.2. The Analytical Parameters of the OSE-Masking ELISA Procedure

**Figure 13:**
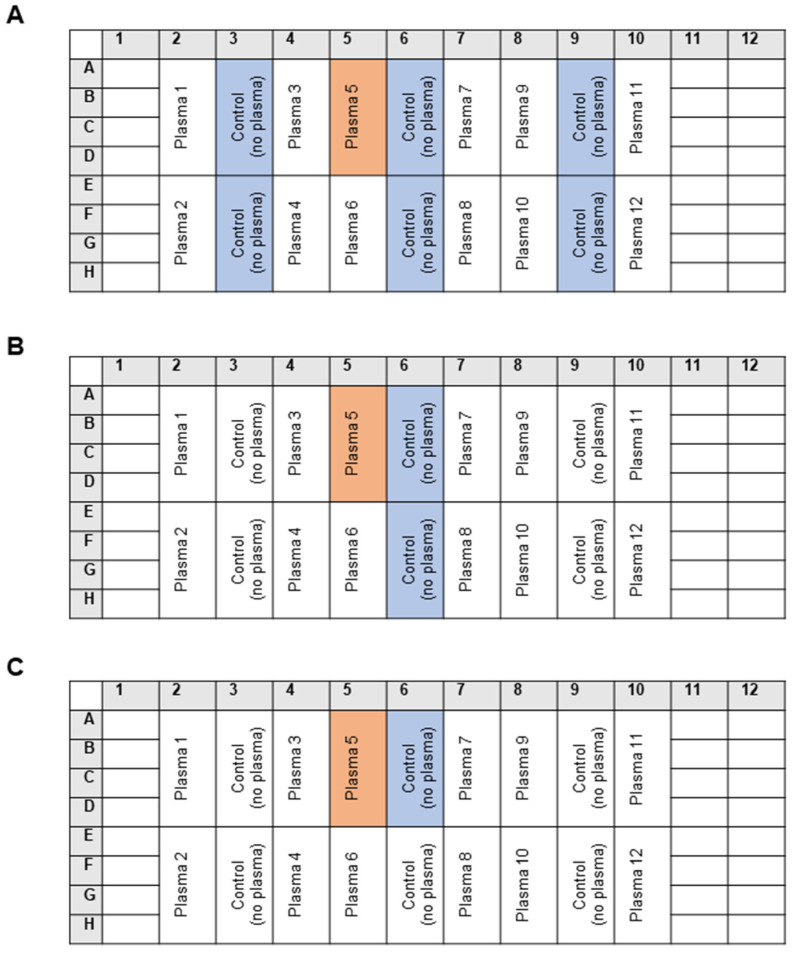
Calculation of the masking activity using three normalization methods. Plasma sample 5 is shown as an example. In approach (**A**), the OD492 values of the wells treated with plasma sample 5 were divided by the mean value of 24 control wells located in columns 3, 6, and 9. Control wells were incubated with PBSE/BSA (10 mg/mL) without plasma. In approach (**B**), the OD492 values of the wells treated with plasma sample 5 were normalized to the mean value of the eight control wells located in adjacent column 6. In approach (**C**), the OD492 values of the wells treated with plasma sample 5 were divided by the mean OD492 of four control wells located in the vicinity of the wells treated with plasma sample 5 (6A, 6B, 6C, and 6D). Normalized values for the other plasma samples were calculated according to the same principle.

The assay was successfully performed in both 96- and 384-well plates. Pipetting of the reagents was performed using multisteppers, single- and multichannel manual or electronic pipettes, and a pipetting robot. All of the pipetting steps produced satisfactory results, with CV values between 2 and 5%. The most common type of experimental bias in immune assays is the intra-plate gradient of the signal. In order to estimate the impact of this source of error, the maximal binding of mAb 509 (i.e., the control samples incubated with plasma-free PBSE/BSA (10 mg/mL)) was determined at several positions (columns 3, 6, and 9) in each 96-well plate (Figure 13). The individual plasma masking capacity was calculated using one of the three approaches presented in Figure 13, namely normalization to (A) the mean of all 24 control wells positioned in columns 3, 6, and 9; (B) the mean of 8 control wells in the column adjacent to an individual sample; or (C) the mean of 4 control wells in the direct vicinity of the sample wells. In order to compare the performance of these methods, we used the results of an OSE-masking ELISA study (results to be published elsewhere) that included samples from 138 subjects that were analyzed in 12 plates on three different days. Each plate included a freshly thawed aliquot of the same pooled plasma sample. We used these pooled plasma values to estimate the statistical variation in the assay. First, the mean OD value of the pooled plasma sample was calculated within each plate. Next, the mean OD values of the no-plasma controls (maximal binding) was calculated using the three approaches described above (24, 8, or 4 control wells). Further, the mean OD value of the pooled plasma was divided by each of the three no-plasma control values, thus producing three masking capacity values for each plate. Finally, the mean masking capacity and CV values were calculated for pooled plasma samples from 12 plates. The data (Table 1) show that the mean values determined using these three methods of normalization were close to each other, thus confirming the lack of intra-plate gradients. The inter-plate CV was close to 10%, which is generally regarded as acceptable.

#### 3.2.1. Intra-Individual Variability in the Masking Activity

Further experiments addressed the short-term day-to-day intra-individual variation in the masking activity. To this end, blood samples were taken from the same donors on five consecutive days. The masking activity of each sample was determined in two clusters of four wells (eight wells per each day of blood sampling) located at two different positions of the plate. All five samples daily collected from each subject were analyzed on a single plate. It was found that in seven of the tested subjects, the variation in the masking activity in the blood samples collected on five consecutive days and quantified in a single assay ranged from 1.8 to 11.2%. The average day-to-day CV in the masking activity in the group was 5.1 ± 2.99%. These values are within the range of inter-plate analytical variation, thus suggesting that the individual masking activity is a stable biological parameter, showing no significant fluctuations within several days.

#### 3.2.2. Inter-Individual Variability in the Masking Activity

**Figure 14:**
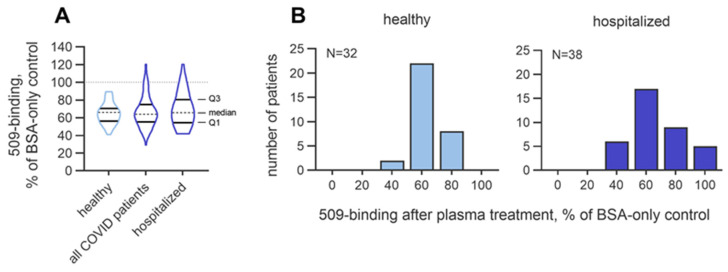
Inter-individual variation in masking activity in SARS-CoV-2-seropositive patients and -seronegative subjects. The masking activity was determined in a group of 98 COVID-19 patients, including 38 who were hospitalized, and a cohort of 32 seronegative subjects. Note that hospitalized patients were characterized by a higher interquartile range (**A**) and higher numbers of subjects with residual mAb 509 binding values below 60% and above 80% (**B**).

Characterizing inter-individual variation is a crucial aspect of assay development. The OSE-masking activity (expressed as the % of the no-plasma control values) in the healthy subjects (n = 32) varied from 41.2 to 89.5%. In addition, assuming that the activity may be influenced by disease, we analyzed the variation in a cohort of SARS-CoV-2-seropositive patients with different disease severities (n = 98) and in a subgroup of patients that were hospitalized (n = 38), of whom 15 had acute respiratory distress syndrome and 9 had sepsis. The OSE-masking activity in the mixed COVID-19 cohort varied from 29.2 to 120%, and in the hospitalized group, it varied from 42.1 to 120%. Although the median values in the three groups were close to each other (66.1, 64.1, and 65.8%, respectively; Figure 14A), it was found that the hospitalized group contained more patients with masking values below 50% and above 80% as compared to these numbers of seronegative subjects (Figure 14B), which pointed to the higher dispersion of the masking activity in the group with severe disease. The interquartile ranges, which quantitatively described the variation within groups, were 14% in the seronegative probands, 19.6% in the combined COVID-19 group, and 26.1% in the hospitalized COVID-19 patients. The trend of increased variation may reflect the heterogeneity of the disease severity and treatment in hospitalized patients. To summarize, these data suggest that acute pathology can influence the variation in masking activity.

## 4. Discussion

Oxidation-specific epitopes (OSEs), including oxidized phospholipids (OxPLs), are increasingly recognized as pathogenic molecules that promote the development of inflammatory and autoimmune diseases [1]. Notably, the pathogenic role of endogenously generated OxPLs has recently been demonstrated in several animal models of disease [7,9,10,23,24]. Furthermore, elevated circulating levels of OxPLs bound to the lipoprotein (a) (Lp(a)) represent a strong risk factor for the development of atherosclerosis in humans [25].

Growing recognition of OxPLs as pathogenic molecules is reflected in the active development of therapeutic approaches aiming to reduce or neutralize this class of OSEs. Experimental therapies that lower the levels or pathogenic activity of OxPLs include the reduction of their major carrier, Lp(a) [25]; the transgenic expression of antibody fragments that neutralize OxPCs [7,8,9,24]; immunization with OxPCs [26]; treatment with apolipoprotein A-derived amphipathic peptides that bind OxPLs [27]; peptides that block the binding of OxPLs to scavenger receptors [28]; and small molecules that support cellular proteostasis [29]. These findings support a potential role of OxPLs as a disease risk factor. However, for an accurate risk assessment, the activity of protective factors may be as important as the absolute concentrations of these lipids. These protective mechanisms remain poorly understood, and reliable methods for their assessment in patient cohorts are largely lacking.

Emerging evidence suggests that pro-inflammatory and pro-atherogenic OSEs are cleared or inactivated in the plasma through enzymatic and protein-binding mechanisms. For example, OxPLs are neutralized enzymatically by lipoprotein-associated phospholipase A_2_ (Lp-PLA2) [12] and non-enzymatically by binding to IgM [13], CRP [17], CFH [18], and other plasma proteins [19]. The current approaches to the analysis of OSE-neutralizing mechanisms in patients’ blood plasma are predominantly limited to determination of OxLDL immune complexes [30] or Lp-PLA2 [31]. However, given the variety of protective mechanisms, a simple test capable of quantifying multiple of such processes is needed. Thus, the key novelty of our approach lies in its ability to detect multiple degradation and neutralization mechanisms within a single assay.

In order to quantitate the OxPL-neutralizing capacity in individual patients, we developed a masking assay based on monoclonal antibodies that recognized OxPCs and OxPEs [20]. In this assay, the binding of mAbs to OSEs serves as a proxy for direct contact between OSEs and pathology-driving receptors, such as complement, scavenger, and lymphocyte receptors. The assay allows for the estimation of how effectively the components of individuals’ plasma degrade OSEs and prevent their interaction with other proteins. The advantage of the masking assay is that its readout is influenced by all of the mechanisms that neutralize OSEs, including enzymatic degradation, as well as OSE masking through the binding of antibodies and non-immunoglobulin proteins.

It is important to emphasize the novelty and differentiation of the masking assay compared to other analytical techniques in this field. Circulating biomarkers of oxidative stress can be measured using several methods, such as quantifying the concentrations of oxidation-specific epitopes (OSEs)—including OxLDL, malondialdehyde (MDA), or OxPL/apoB—via immunoassays [1,25,32,33] or detecting oxysterols and OxPLs through mass spectrometry [34,35,36,37]. Additionally, some assays evaluate antibodies of specific classes that recognize OSEs, such as IgM-OxLDL or IgG-OxLDL immune complexes [38]. The key difference in the masking assay lies in its focus—not on quantifying the concentration of OSEs but on assessing the activity of neutralizing mechanisms, such as OSE degradation or the formation of a protein “corona” that prevents their interaction with other proteins or receptors. In this work, we focused on a specific type of OSE, namely OxPE, but essentially, the same procedure can quantify masking of other classes of OxPLs and other structural types of OSEs with the help of the corresponding mAbs. We used OxLDL as a known source of the OxPLs which are present inside the lipoprotein particle in a more natural environment compared to pure synthetic OSEs. However, the same assay could be performed using individual pure OSEs, such as adducts of specific LPPs with individual proteins, e.g., MDA-BSA.

We tested various experimental parameters of the assay and made several observations that are practically relevant to conducting clinical studies aiming to identify potential clinical associations. Notably, we identified several compounds that interfered with the masking assay, including the commonly used anticoagulant heparin. On the positive side, we observed sufficient pre-analytical stability of the activity, which is essential for a parallel analysis of multiple samples. Additionally, we found significant intra-individual stability in the masking activity when measured over five consecutive days. These data suggest that its activity does not undergo acute fluctuations that would undermine the reliability of multiple measurements within an individual.

In summary, the masking assay is a simple and affordable immunological method for estimating anti-OSE-protective mechanisms in human blood plasma. In this manuscript, we focused on the OxPE type of OSE, but this study also described general factors influencing the assay, making it a starting point for establishing masking assays for other types of OSEs. An analysis of the masking activity in patients may support the stratification of individual risk and therapeutic approaches to lipid-peroxidation-related diseases. Future work in different at-risk populations and patient groups, including the effects of pharmacologic interventions, will demonstrate the opportunities and limitations of the assay in clinical settings.

## Figures and Tables

**Table 1:** The effect of the normalization method on inter-plate mean values and CVs.

	Masking Activity of Pooled Plasma Normalized to		
	Meansof 3 × 8 Control Wellsin Columns 3, 6 and 9	Meansof 1 × 8 Control Wellsin Column 9	Meansof 4 Vicinal Control Wellsin Column 9
Mean of 12 plates	0.58	0.62	0.64
SD	0.06	0.06	0.06
CV, %	10.9	10.4	9.2

Identical pooled plasma was applied in each of 12 plates to wells E10-H10. The mean OD value of the pooled plasma was normalized to the control plasma-free samples. In this test, the mean signal of the pooled plasma in each plate was normalized to (i) the mean of 24 control (no-plasma) wells (columns 3, 6, and 9), (ii) the mean of 8 control wells in column 9 (the column adjacent to the pooled plasma wells), or (iii) the mean of 4 control wells in column 9 vicinal to the pooled plasma wells (Figure 13).

## Data Availability

No datasets were generated during this study.

## Abbreviations

The following abbreviations are used in this manuscript:

apoB	Apolipoprotein B-100
BSA	Bovine serum albumin
C1q	Complement C1q subcomponent
CFH	Complement factor H
CRP	C-reactive protein
DAMPs	Damage-associated molecular patterns
FcR	Fc-receptor
IgM	Immunoglobulins of the M class
Lp-PLA2	Lipoprotein-associated phospholipase A2
Lp(a)	Lipoprotein (a)
LPPs	Lipid peroxidation products
mAb	Monoclonal antibody
MDA	Malondialdehyde
OSE	Oxidation-specific epitope
OxLDL	Oxidized low-density lipoprotein
OxPAPC	Oxidized palmitoyl-arachidonoyl-phosphatidylcholine
OxPC	Oxidized phosphatidylcholine
OxPLs	Oxidized phospholipids
OxPE	Oxidized phosphatidylethanolamine
PBS	Phosphate-buffered saline
PBSE	Phosphate-buffered saline supplemented with EDTA
